# Early and late effects of Ibuprofen on mouse sperm parameters, chromatin condensation, and DNA integrity in mice

**Published:** 2015-11

**Authors:** Fatemeh Roodbari, Nahid Abedi, Ali Reza Talebi

**Affiliations:** 1 *Department of Biology, Faculty of Basic Sciences, University of Mazandaran, Sari, Iran.*; 2 *Department of Biology and Anatomical Sciences, Research and Clinical Center for Infertility, Shahid Sadoughi University of Medical Sciences, Yazd, Iran*

**Keywords:** *Ibuprofen*, *Sperm*, *DNA*, *Chromatin*, *Mice*

## Abstract

**Background:**

There are few studies indicating the detrimental effects of ibuprofen on sperm fertility potential and DNA integrity.

**Objective:**

To determine the effects of Ibuprofen on sperm parameters, chromatin condensation and DNA integrity of mice.

**Materials and Methods:**

In this experimental study, 36 adult male mice with average weight 37 gr were divided into three groups, including control (group I, n=12), normal dosage of ibuprofen (group II, n=12) and high dosage (group III, n=12). Ibuprofen with different doses was dissolved in daily water of animals. After 35, 70 and 105 days, the cauda epididymis of mice were cut and incubated in Ham’s F10 media. Sperm samples were analyzed for parameters (motility, morphology and count), DNA integrity (SCD test) and chromatin condensation (chromomycin A3 and Aniline blue staining).

**Results:**

After 35 days, in addition to above mentioned sperm parameters, all of the treated mice showed statistically significant increase in spermatozoa with immature chromatin (P<0.05). However, after 70 days, the rate of sperm DNA fragmentation assessed by SCD was increased in group II (66.5±0.7) and the percentage of immature spermatozoa (AB^+^ and CMA3^+^) was higher in group III (77.5±0.7 and 49.5±6.3 respectively) than other groups. After 105 days, the AB^+^ spermatozoa were increased in both normal dose and high dose groups.

**Conclusion:**

Ibuprofen may cause a significant reduction in sperm parameters and sperm chromatin/DNA integrity in mice. It should be noted that these deleterious effects are dose-dependent and can be seen in early and late stage of drug treatments.

## Introduction

Infertility is a major problem for 15-20% of young couples and 50% of cases are related to male factors (1). The causes of some male infertility have been identified such as gene mutant, aneuploidies, infections, diseases, varicocele, radiation, drugs and so on. On the other hand, in many cases the etiology of male infertility is not clear (2), so the study about life styles and several habits, which can give rise to male infertility are important. The quality of sperm DNA is very important in the mammalian reproductive potential (3). The sperm cell transmits the paternal genetic codes to the embryo (4). So, the men with normal sperm motility and morphology may be still infertile, due to the owning abnormal sperm DNA (3). In fact, the accurate transfer of genome depends on sperm chromatin structure (2), as a result of facing with chemical and physical agents during transfer in both sexes genital tracts (5). So, sperm cell DNA needs to be denser and more organized than somatic one (6). In this structure, histones are replaced by basic protamines, which are smaller and have more positive charge than histones.

In addition, the genetic material in male sperm has a critical role in embryonic development specially during the first days (7) and it is an important factor in successful assisted reproductive treatment (ART). Tesarik *et al*. suggested sperm DNA fragmentation can cause Intra-cytoplasmic Sperm Injection failure (8). Moreover, Borini *et al.* have shown that the paternal genome not only has strong effect on fertilization and embryo quality, but also affects embryo viability (9). 

There are several factors that menace sperm DNA sperm integrity. Pathological and environmental factors like varicocele, alcohol, narcotics and some drugs such as ibuprofen could affect sperm DNA structure and chromatin condensation (10). These painkiller drugs are available without prescription in several parts of the world and many people use them real vast without paying attention to their dosage and probable side effects (11). 

Ibuprofen is a propionic acid and one of the non-steroidal anti-inflammatory drugs (NSAD_S_) that has both analgesic and antipyretic effects (12). Ibuprofen and other NSADs are ingested for many reasons like fevers, cold, flu, general pains, and rheumatoid arthritis. These drugs inhibit the activity of cyclooxygenases (COX1 and COX2), which synthesize and release prostaglandins (PGs) (13, 14). Although, the PGs have many diverse roles in the most tissues, but, their roles in spermatogenesis are not well known. Templeton et al. have shown that prostaglandin E, 19-hydroxy prostaglandin E, prostaglandin F, and 19-hydroxy prostaglandin are present in human semen, but they did not explain their roles. It was shown that PGs come from prostate (15). However, the study of Fang et al. has indicated that the prostaglandin D synthase has a key role in the regulation of spermatogenesis in Chines mitten crab Eriocheirsinensis (16).

The aim of present study was to investigate the effects of ibuprofen on sperm parameters, chromatin condensation, and DNA integrity using cytochemical based (Aniline blue and Chromomycin A3) and molecular based (sperm chromatin dispersion) assays in mice. 

## Materials and methods

**Animals**

This experimental study was done in Research and Clinical Center for Infertility, Yazd, Iran from September to December of 2014. Totally 36 adult male NMRI mice with average weight of 37 g and 11 weeks old were obtained from the mouse stock of Shahid Sadoughi University of Medical Sciences. These mice were divided into three groups; control (group I, n=12), normal dosage (group II, n=12) and high dosage (group III, n=12). In groups II and III, 30 and 57 mg/kg body weight ibuprofen were dissolved in daily drink water, respectively (10), but, in the control group, we did not have any medication. During experiment, animals were kept in standard conditions with a temperature range of 25±3^o^C and mean relative humidity of 50±5% in the animal house. During the course of study, it was followed the recommendations by our Institutional Animal Care and Use Committee for the handling, maintenance, treatment, and killing of the animals. This experimental project was approved by the Ethical Committee of Yazd Research and Clinical Center for Infertility, Shahid Sadoughi University of Medical Sciences.

**Epididymal sperm preparation**

The duration of spermatogenesis in mouse is about 35 days (17). We assessed the spermatozoa of drug-treated mice after 1, 2 and 3 durations of spermatogenesis in each group. In other word, the sperm samples were analyzed on 35, 70 and 105 days post treatments. The mice in each groups were killed by cervical dislocated and the cauda epididymis of each animal was cut and placed in 1 ml Ham’s F10 medium. The dishes were incubated at 37 and 5% CO_2_ for 10 min to make spermatozoa swim out (18).

**Sperm parameters analysis**

The sperm motility, normal morphology, viability and sperm count were evaluated for at least 200 spermatozoa of each animal. Sperm movement analysis was done by Makler chamber and light microscopy at x 40 eyepiece magnification (Olympus Co., Tokyo, Japan). Motility was expressed as the percentages of progressive motility, including progressive (Rapid and Slow), non-progressive and immotile spermatozoa. The morphologically normal spermatozoa and the percentage of viable sperm cells were assessed by Papanicolaou staining and Eosin test, respectively (19). The light microscope was set at × 100 eyepiece magnification.

**Sperm chromatin and DNA evaluations**

Sperm chromatin condensation and DNA integrity were assessed using Sperm Chromatin Dispersion (SCD) test, chromomycin A3 (CMA3) and Aniline blue (AB) staining.

**1. Aniline blue**

Aniline blue staining indicates the excessive histones in chromatin structure. After smearing the epididymal spermatozoa, the slides were air-dried and then fixed in 3% buffered glutaraldehyde in 0.2 M phosphate buffer (pH 7.2) for 30 min at room temperature. Each smear was stained with 5% aqueous AB stain (Sigma, St Louis, MO, USA) in 4% acetic acid (pH 3.5) for 15 min (18). 

**2. Chromomycin A3 staining**

CMA3 is a fluorochrome antibiotic, which competes with the protamines for binding to the minor groove of DNA (6). The air-dried smears were fixed with Carnoy's solution at 4 °C for 10 min. Then, each slide was stained with 500 µl of CMA3 solution (50 µl stock stain solution + 450 µl McIlvaine buffer) (Sigma, St Louis, MO, USA) in darkroom for 20 min (20).

**3. SCD test**

The SCD test was used for the assessment of sperm DNA fragmentation. The SCD test was performed using the Halosperm® Kit (INDAS laboratories, Madrid, Spain). For the mouse sperm staining, after adding 25 μl of semen to 50 μl of low melting agarose, 10 μl of this suspension was decanted on coated slide of Halosperm® Kit. A small lamella was put on it and kept on 4°C for 5 min. The slide was immersed in Denaturant Agent (for 7min) and Lysis Solution (for 20 min). After dehydrating, the slides were stained with Staining Solution A & B for 7 min (20).

**Statistical analysis**

All statistical analyses were performed by using Statistical Package for the Social Sciences, version 20.0, SPSS Inc, Chicago, Illinois, USA (SPSS 20) software. Data were expressed in mean±SD (standard deviation). One-way ANOVA followed by Tukey-HSD and Pearsons´ correlation test was considered for comparison of the results and the term 'statistically significant' was used for p-value less than 0.05.

## Results

Table I indicates the results of semen analysis for sperm parameters. Some of the parameters such as the numbers of sperm cells with normal morphology, and viability (except for group III) were changed with only one period of drug treatment (35 days). While, some other parameters like sperm count were revealed significant changes after ongoing use of the drug for two periods of spermatogenesis (70 days). Although, some of the parameters showed significant variation with a continuous use of drug such as progressive motility, but some of them did not change. The variables were perused during three times of exposure to drugs in each group. Also, the number of non-progressive motile spermatozoa in group III, which were received 35 days drug were increased compared to the same group after receiving drug in 70 and 105 days (p value=0.011 and 0.022, respectively). Total motility of sperm had significant decrease in mice that were treated 35 mg/kg/day for 70 days (p value=0.001).

Table II shows the results of sperm chromatin analysis. The results of samples that were treated for one period of spermatogenesis were positive for all three sperm chromatin tests. While these results for other samples were different. After 105 days of drug treatment, the result of DNA assessment did not show increasing in DNA fragmentation and protamine deficiency (CMA3 staining). In this study, it was used Pearsons´ correlation test to find out the correlation between the variables. The results with p value less than 0.05 of this test are as follows:

• The correlation between progressive motility and count (p-value<0.001and Pearsons' correlation= 0.816).

• Progressive motility and viability (p-value< 0.001 and Pearsons' correlation= 0.816).

• Progressive motility and Aniline blue staining (p-value= 0.007 and Pearsons' correlation= -0.638).

• Progressive motility and normal morphology (p-value= 0.002 and Pearsons' correlation= 0.400).

• DNA fragmentation (SCD test) and abnormalities in sperm tail (p-value= 0.018 and Pearsons' correlation= 0.363).

• Normal morphology and protamine deficiency (CMA3 staining) (p value= 0.029 and Pearson correlation= -0.337).

• Normal morphology and DNA fragmentation (SCD test) (p-value= 0.004 and Pearson correlation= -0.436).

• Protamine deficiency and DNA fragmentation (p-value= 0.050 and Pearsons' correlation= 0.304).

- Protamine deficiency and Aniline blue staining (p-value= 0.021 and Pearsons' correlation= -0.354).

**Table I:** The difference in mouse sperm parameters between control and case groups

**Variables**	**The time of exposure to drug**	**Group I** **(Control group)**	**Group II** **(30 mg/kg/day Ibuprofen)**	**Group II** **(57 mg/kg/day Ibuprofen)**	**P-value**
**Count (×10** ^6^ **)**	One period of spermatogenesis	10.5±0.7	6.0±2.8	7.5±0.7	0.140^a^ 0.118^b^ 0.678^c^
	Two periods of spermatogenesis	10.0±1.0	7.0±1.0	6.6±0.5	0.002^a^ 0.001^b^ 0.997^c^
	Three periods of spermatogenesis	10.7±0.5	4.2±0.5	4.7±0.5	0.000^a^ 0.000^b^ 0.995^c^
**Progressive motility (%)**	One period of spermatogenesis	35.5±2.1	13.5±2.1	23.5±4.9	0.000^a^ 0.002^b^ 0.994^c^
	Two periods of spermatogenesis	34.6±1.1	10.6±1.5	16.6±1.1	0.000^a^ 0.000^b^ 0.234^c^
	Three periods of spermatogenesis	53.3±4.1	5.3±0.5	2.6±2.5	0.000^a^ 0.000^b^ 0.997^c^
**Non progressive motility (%)**	One period of spermatogenesis	27.5±3.5	23.5±2.1	29.0±1.41	0.84^a^ 0.99^b^ 0.613^c^
	Two period of spermatogenesis	26.0±1.0	51.3±3.2	36.6±0.57	0.000^a^ 0.017^b^ 0.001^c^
	Three period of spermatogenesis	21.2±2.4	52.5±7.0	46.7±3.9	0.000^a^ 0.000^b^ 0.691^c^
**Immotile sperm (%) **	One period of spermatogenesis	I. 37.0±2.8	I. 63.0±0.0	I. 47.5±6.3	0.002^a^ 0.155^b^ 0.029^c^
	Two periods of spermatogenesis	II.39.3±0.05	II. 38.0±0.99	II. 46.6±0.5	0.990^a^ 0.180^b^ 0.081^c^
	Three periods of spermatogenesis	III. 30.75±2.0	III. 42.2±6.5	III.50.5±5.1	0.074^a^ 0.001^b^ 0.340^c^
**Total motility (%) **	One period of spermatogenesis	63±1.41	37.0±0.00	57.0±0.70	0.027^a^ 0.951^b^ 0.083^c^
	Two periods of spermatogenesis	60.66±0.57	62.00±1.7	53.3±0.5	0.990^a^ 0.000^b^ 0.083^c^
	Three periods of spermatogenesis	69.25±2.0	58.75±4.5	49.5±5.1	0.900^a^ 0.002^b^ 0.173^c^
**Normal morphology**	One period of spermatogenesis	57.5±3.5	31.5±0.7	29.0±1.4	0.000^a^ 0.000^b^ 0.886^c^
	Two periods of spermatogenesis	58.0±3.4	32.3±2.5	23.6±4.6	0.000^a^ 0.000^b^ 0.181^c^
	Three periods of spermatogenesis	58.0±3.4	42.0±10.6	36.0±2.0	0.300^a^ 0.002^b^ 0.840^c^
**Viability**	One period of spermatogenesis	71.5±0.7	6.0±2.8	56.0±8.4	0.146^a^ 0.041^b^ 0.920^c^
	Two periods of spermatogenesis	79.6±5.0	63.6±8.0	65.3±0.5	0.016^a^ 0.001^b^ 0.999^c^
	Three periods of spermatogenesis	70.2±0.9	57.0±5.0	60.2±1.5	0.028^a^ 0.160^b^ 0.974^c^

* Standard's one way ANOVA test (all data are presented as mean±SD)

a : Difference between group I and II;

b : Difference between group I and III;

c : Difference between case groups

**Table II:** The difference in mouse sperm chromatin assessment between control and case groups

**Variables**	**The time of exposure to drug**	**Group I** **(Control group)**	**Group II** **(30 mg/kg/day Ibuprofen)**	**Group III** **(57 mg/kg/day Ibuprofen)**	**P-value**
**AB staining**	One period of spermatogenesis	59.0±1.4	66.5±0.7	77.5±0.7	0.007^a^ 0.000^b^ 0.103^c^
	Two periods of spermatogenesis	57.0±15.5	74.5±6.3	75.0±12.7	0.450^a^ 0.004^b^ 0.974^c^
	Three periods of spermatogenesis	51.7±0.5	73.0±2.4	64.7±1.2	0.000^a^ 0.000^b^ 0.001^c^
**CMA 3 staining**	One period of spermatogenesis	22±2.8	52.5±3.5	49.5±6.3	0.016^a^ 0.027^b^ 0.998^c^
	Two periods of spermatogenesis	29.0±9.8	51.5±4.9	68.0±11.3	0.210^a^ 0.020^b^ 0.476^c^
	Three periods of spermatogenesis	43.0±15.5	62.0±2.8	89.5±7.7	0.620^a^ 0.310^b^ 0.990^c^
**SCD**	One period of spermatogenesis	52.5±3.5	66.5±0.7	77.5±0.7	0.007^a^ 0.000^b^ 0.090^c^
	Two periods of spermatogenesis	48.0±1.4	75.0±12.7	74.0±15.5	0.004^a^ 0.004^b^ 1.000^c^
	Three periods of spermatogenesis	50.0±0.0	63.0±14.1	54.5±6.3	0.517^a^ 0.991^b^ 0.849^c^

* standard's one way ANOVA test (all data were presented as mean±SD)

a :Difference between groups I and II; ;

b : Difference between groups I and III

c : Difference between case groups

Ab : Aniline Blue, CMA3: Chromomycin A3; SCD: Sperm Chromatin Dispersion

## Discussion

PGs are one of the important chemical mediators in the body, which were firstly isolated from seminal fluid in 1935 (21). They are present in the most tissues and play an important role in most of inflammations. Studies have indicated that PGA, PGB, PGH, 19-OH PGA, 19-OH PGB and 19-OH PGE exist in seminal fluid of fertile men (22, 23). On the other hand, reduced seminal PGs was observed in infertile seminal fluid that had idiopathic infertility (23). In male reproductive system, PGs are secreted by seminal vesicle and prostate glands (24, 25) and have two different functions: firstly, they increase sperm progressive motility (25, 26) and secondary, they help the sperm penetration into the ovum (22). In the present study, for the first time, it was shown the impacts of Ibuprofen on sperm parameters and DNA integrity in mouse as an experimental model. The assessment of sperm motility indicated that Ibuprofen can reduce the percentage of quick spermatozoa motility in both case groups on early and late stages. These results were in accordance with Ekalou *et al*. who studied the negative effects of aspirin as a non-steroidal inhibitor of cyclo-oxygenase (COX) on sperm motility (26). Decreasing in sperm normal morphology showed a different pattern. Although, it was observed a significant decline in normal sperm in groups II and III after 35 and 70 days post-treatment, but this decrease was observed only in group III after 105 days. It seems that using drug has prominent deleterious effects on the late stages. Ekalou et al. also observed similar results after 90 days treatments of aspirin (26). The reduction in sperm count and viability may be the result of cell death by necrosis or apoptosis, which was induced by drug treatments especially in high dose group on late stages.

One of the important sign of cell death is chromatin and DNA abnormalities. Sperm chromatin condensation was assessed using aniline blue staining showed a reduction in group II after 35 and 70 days and in group III after three periods of time. It was clear that mice who received 57 mg/kg/day Ibuprofen had more spermatozoa with lower condensed chromatin than group II who received 30 mg/kg/day ibuprofen.

The result of Pearsons´ correlation test between sperm DNA fragmentation (SCD test), count and normal morphology indicated that DNA fragmentation, morphology and count were related to each other. It confirmed the idea that morphologically normal spermatozoa have better DNA and chromatin quality and they can be used for ART. Moreover, Lee *et al*. (2002) have shown that sperm morphology is a good criterion for male fertility (27). Our study also indicated that sperm normal morphology had a positive correlation with sperm motility. 

Nuclear protamine deficiency (CMA3 staining) of spermatozoa was detected in both groups II and III after 35 days and was continued in group III after 70 days, but it was returned after 105 days.

Surprisingly, after 105 days protamine deficiency and the number of spermatozoa with morphological abnormality were decreased. This implied that longitudinal use of ibuprofen may reduce the inhibiting effect on COX or the cell might be synthesizing more COX enzymes. It seems that in addition to the effect of PGs on motility and morphology of spermatozoa, they also might affect the integrity of sperm DNA. However, there are no study that indicate the impact of Ibuprofen on sperm chromatin and DNA integrity.

PGs are made by the enzyme - COX from arachidonic acid (AA). COX has two different isoforms, including COX1 and COX2 (28, 29). COX1 exists in intestinal mucosa, platelets, endothelial cells of vessels and kidney while COX2 is present during inflammatory responses (30). COX is a homodimer, which has a PG hydroxylase catalytic domain and also cyclo-oxygenases catalytic domain. COX moreover, has a third domain that is bound to sER membrane, which is binding domain. Two catalytic domains of COX enzyme are near together and the binding of one substrate or inhibitor to one catalytic domain effects on another catalytic domain (31). Non-steroidal anti-inflammatory drugs like ibuprofen inhibit COX by noncovalent binding and lead to decreasing PGs synthesis. COX has two kinds of substrate for producing PGs, including AA and 2-arachidonic acid (2AG). COX synthesizes PGs by AA and PG-Gs by 2AG. 

Ibuprofen inhibits COX in two different ways when each substrate is available. When COX used, AA as substrate to make PG, Ibuprofen inhibits it uncompetitive. It occupies one active site then provides binding substrate to other active site. But, if 2AG is substrate, binding Ibuprofen to one active site is necessary, but it is not sufficient. It needs more construction of Ibuprofen to inhibit other active site. This is a competitive inhibitor (31, 32). However, Ibuprofen decreases PG synthesizes with inhibition of COX enzyme, especially COX2 (31).

## Conclusion

In conclusion, the present study showed that Ibuprofen may cause a significant reduction in sperm parameters (motility, morphology and count) and sperm chromatin/DNA integrity in mice. It should be noted that these deleterious effects are dose-dependent and can be seen in early and late stages of drug treatments.

## Note

***This article is extracted from M.Sc. thesis. (Nahid Abedi)***

## References

[1] Hekmatdoost A, Lakpour N, Sadeghi MR (2009). Sperm chromatin integrity: etiologies and mechanisms of abnormality, assays, clinical importance, preventing and repairing damage. Avicenna J Med Biotechnol.

[2] Agarwal A, Said TM (2003). Role of sperm chromatin abnormalities and DNA damage in male infertility. Hum Reprod Update.

[3] Ashwini LS, Javed A, Muralidhar TS, Nagraj R (2015). Commentary: Assessment of an assortment of factors on DNA damage veracity of humansperm. The Pharma Innovation Journal.

[4] Castillo J, Estanyol JM, Ballesc_E JL, Oliva R (2015). Human sperm chromatin epigenetic potential: genomics, proteomics, and male infertility. Asian J Androl.

[5] Ward WS, Coffey DS (1991). DNA packaging and organization in mammalian spermatozoa: comparison with somatic cells. Biol Reprod.

[6] Iranpour FG, Nasr Esfahani MH, Valojerdi MR, Al-Taraihi TMT (2000). Chromomycin A3 staining as a useful tool for evaluation of male fertility. J Assist Reprod Genet.

[7] Wdowiak A, Bakalczuk S, Bakalczuk G (2015). The effect of sperm DNA fragmentation on the dynamics of the embryonic development in intracytoplasmatic sperm injection. Reprod Biol.

[8] Tesarik J, GrecoE, Mendoza C (2004). Late, but not early, paternal effect on human embryo development is related to sperm DNA fragmentation. Hum Reprod.

[9] Borini A, Tarozzi N, Bizzaro D, Bonu MA, Fava L, Flamigni C (2006). Sperm DNA fragmentation: paternal effect on early post-implantation embryo development in ART. Hum Reprod.

[10] Tripathi R, Pancholi SS, Tripathi P (2012). Genotoxicity of ibuprofen in mouse bone marrow cells in vivo. Drug Chem Toxicol.

[11] SAHU CR (2009). Developmental Toxicity Of Ibuprofen Treated Mice. Int J Pharm Pharm Sci.

[12] Elli M, Gaffuri B, Frigerio A, Zanardelli M, Covini D, Candiani M (2001). Effect of a singledose of ibuprofen lysinate before embryo transfer on pregnancy rates in cows. Reproduction.

[13] Omar TY (2014). The Effect of ibuprofen and vitamin c on some blood parameters and the reproductive organs of the male rabbits. World Journal Of Pharmacy And Pharmacutical Sciences.

[14] Orlando BJ, Lucido MJ, Malkowski MG (2015). The structure of ibuprofen bound to cyclooxygenase-2. J Struct Biol.

[15] Templeton AA, Cooper I, Kelly RW (1978). Prostaglandin concentrations in the semen of fertile men. J Reprod Fertil.

[16] Fang DA, Yang QZ, Duan JR, Wang Q, Zhang MY, Zhou YF (2014). Characteristic of PGDS potential regulation role on spermatogenesis in the Chinese mitten crab Eriocheir sinensis. Gene.

[17] Petropoulos S, Matthews SG, Szyf M (2014). Adult glucocorticoid exposure leads to transcriptional and DNA methylation changes in nuclear steroid receptors in the hippocampus and kidney of mouse male offspring. Biol Reprod.

[18] Talebi AR, Sarcheshmeh AA, Khalili MA, Tabibnejad N (2011). Effects of ethanol consumption on chromatin condensation and DNA integrity of epididymal spermatozoa in rat. Alcohol.

[19] Mangoli E, Talebi AR, Anvari M, Pourentezari M (2013). Effects of experimentally-induced diabetes on sperm parameters and chromatin quality in mice. Iran J Reprod Med.

[20] Rahimipour M, Talebi AR, Anvari M, Abbasi A (2014). Saccharin consumption increases sperm DNA fragmentation and apoptosis in mice. Iran J Reprod Med.

[21] Goldblatt MW (1935). Properties of human seminal plasma. J Physiol.

[22] Aitken RJ, Kelly RW (1985). Analysis of the direct effects of prostaglandins on human sperm function. J Reprod Fertil.

[23] Templeton AA, Cooper I, Kelly RW (1978). Prostaglandin concentrations in the semen of fertile men. J Reprod Fertil.

[24] Gerozissis K, Jouannet P, Soufir JC, Dray F (1982). Origin of prostaglandins in human semen. J Reprod Fertil.

[25] Gonzales GF (2001). Function of seminal vesicles and their role on male fertility. Asian J Androl.

[26] Ekaluo UB, Ikpeme EV, Udokpoh AE (2012). Sperm head abnormality and mutagenic effects of aspirin, paracetamol and caffeine containing analgesics in rats. Int J Toxicol.

[27] Lee RK-K, Hou J-W, Ho H-Y, Hwu Y-M, Lin M-H, Tsai Y-C (2002). Sperm morphology analysis using strict criteria as a prognostic factor in intrauterine insemination. Int J Androl.

[28] Hu S, Bradshaw HB, Chen JS, Tan B, Walker JM (2008). Prostaglandin E2 glycerol ester, an endogenous COX2 metabolite of 2-arachidonoylglycerol, induces hyperalgesia and modulates NFkB activity. Br J Pharmacol.

[29] Dang CT, Shapiro CL, Hudis CA (2002). Potential role of selective COX-2 inhibitors in cancer management. Oncology.

[30] Zarghi A, Arfaei S (2011). Selective COX-2 inhibitors: a review of their structure-activity relationships. Iran J Pharm Res.

[31] Prusakiewicz JJ, Duggan KC, Rouzer CA, Marnett LJ (2009). Differential sensitivity and mechanism of inhibition of COX-2 oxygenation of arachidonic acid and 2-arachidonoylglycerol by ibuprofen and mefenamic acid. Biochemistry.

[32] Lanza FL, Rack MF, Simon TJ, Quan H, Bolognese JA, Hoover ME (1999). Specific inhibition of cyclooxygenase-2 with MK-0966 is associated with less gastroduodenal damage than either aspirin or ibuprofen. Aliment Pharmacol Ther.

